# Taurine supplementation reduces oxidative stress and protects the liver in an iron-overload murine model

**DOI:** 10.3892/mmr.2014.2544

**Published:** 2014-09-05

**Authors:** ZEYU ZHANG, DAN LIU, BO YI, ZHANGPING LIAO, LEI TANG, DONG YIN, MING HE

**Affiliations:** 1 State Key Laboratory of Food Science and Technology, Nanchang University, Nanchang, Jiangxi 330047, P.R. China; 2 Department of Pharmacology and Molecular Therapeutics, Nanchang University School of Pharmaceutical Science, Nanchang, Jiangxi 330006, P.R. China; 3 Second Abdominal Surgery Department, Jiangxi Province Tumor Hospital, Nanchang, Jiangxi 330029, P.R. China; 4 Jiangxi Provincial Key Laboratory of Molecular Medicine at the Second Affiliated Hospital, Nanchang University, Nanchang, Jiangxi 330006, P.R. China

**Keywords:** taurine, iron overload, liver, oxidative stress, apoptosis

## Abstract

We previously demonstrated that iron overload induces liver damage by causing the formation of reactive oxygen species (ROS). Taurine is a potent free radical scavenger that attenuates the damage caused by excessive oxygen free radicals. Therefore, the aim of the present study was to investigate whether taurine could reduce the hepatotoxicity of iron overload with regard to ROS production. Mice were intraperitoneally injected with iron 5 days/week for 13 weeks to achieve iron overload. It was found that iron overload resulted in liver dysfunction, increased apoptosis and elevated oxidative stress. Taurine supplementation increased liver taurine levels by 40% and led to improved liver function, as well as a reduction in apoptosis, ROS formation and mitochondrial swelling and an attenuation in the loss of the mitochondrial membrane potential. Treatment with taurine mediated a reduction in oxidative stress in iron-overloaded mice, attenuated liver lipid peroxidation, elevated antioxidant enzyme activities and maintained reduced glutathione levels. These results indicate that taurine reduces iron-induced hepatic oxidative stress, preserves liver function and inhibits hepatocyte apoptosis. Therefore, taurine may be a potential therapeutic drug to reduce liver damage caused by iron overload.

## Introduction

Secondary iron overload and primary hemochromatosis have received increasing attention due to worldwide epidemics (1–3). Iron is deposited in numerous tissues, most notably the liver, where it can lead to significant organ damage (4). Iron overload in the liver is observed in patients with chronic liver diseases, including alcoholic liver disease and chronic viral hepatitis, and is a secondary side effect of repeated blood transfusions (5–7). Emerging evidence suggests that oxidative stress, mediated by free radicals and reactive oxygen species (ROS), may have a role in the pathophysiology of iron-induced liver injury and cell death (8,9). In a previous study we found that iron overload initiates excessive ROS formation, and that the liver is severely damaged as a result of this oxidative stress (10). However, little is known about the use of antioxidants as possible preventive or curative agents in excessive iron-induced liver damage.

Taurine, a sulfur-containing amino acid, is present at high concentrations in the liver (11). Numerous studies have found that taurine has a protective effect against chemically-induced hepatotoxicity (12–15). Furthermore, taurine has been reported to act as an antioxidant in biological systems. As an antioxidant, taurine has the ability to scavenge ROS and attenuate lipid peroxidation and, as a consequence, stabilizes biological membranes (16,17).

Since taurine has been shown to have potent antioxidant properties, its role in hepatotoxicity induced by iron overload was investigated in the present study. The status of the physiological parameters associated with hepatotoxicity induced by iron overload, including the levels of intracellular antioxidant enzymes, the glutathione (GSH)/glutathione disulfide (GSSG) ratio and the involvement of the mitochondria-dependent apoptotic pathway, was assessed, either with or without supplementation of taurine. The results of this may provide critical information for the treatment of oxidative stress mediated by iron overload in the liver.

## Materials and methods

### Experimental animals and iron overloading protocols

Forty-eight male Kunming mice, weighing 14.07±0.7 g, were obtained from Nanchang University (Nanchang, China). The mice were randomly divided to receive either taurine (0.1 mol/l) or vehicle (2.5% dextrose) in their drinking water 2 weeks prior to the initial injections and throughout the course of the experiments. The mice were continuously injected with either iron or placebo (0.1 ml 10% dextrose) for a total of 13 weeks (total dose, ~200 mg/25 g body weight) as previously described (18). Mice were thus divided into the placebo plus vehicle, placebo plus taurine, iron plus vehicle and iron plus taurine groups. All care and experiments in this study conformed to the National Institutes of Health (NIH) Guide for Care and Use of Laboratory Animals (NIH publication 86–23, revised in 1986). The use of animals was reviewed and approved by the Nanchang University Animal Care Review Committee. At the end of the experiment the mice were sacrificed by cervical dislocation and blood was collected by cardiac puncture. The liver was immediately excised, weighed and divided for further analysis.

### Determination of iron levels in the serum and liver

The iron concentration in the serum was determined using an assay based on the generation of an iron-ferrozine complex, as described by Galleano and Puntarulo (19). The iron concentration in the digested liver sample was measured using a spectrophotometer (535 nm) following the addition of 2 mM bathophenanthroline disulfonic acid.

### Determination of aspartate transaminase (AST) and alanine transaminase (ALT) levels

Serum levels of AST and ALT were measured using an autoanalyzer (Roche Cobas Integra 400, Roche Diagnostics, Holliston, MA, USA) and AST and ALT reagent kits from Roche Diagnostics (Indianapolis, IN, USA).

### Determination of taurine levels

The liver tissue was homogenized and the supernatant was ultra-filtered and diluted with methionine sulfone (an internal standard). The taurine concentration was determined as previously described using a high-performance liquid chromatography system and a specific Pico-Tag column (Waters Corp., Milford, MA, USA) (20).

### Terminal deoxynucleotidyl transferase-mediated dUTP nick end labeling (TUNEL) assay

The TUNEL assay was performed using a commercially available kit (Promega Corp., Madison, WI, USA) in accordance with the manufacturer’s instructions. Briefly, liver samples were fixed by perfusion with 10% buffered formalin, and 5-μm paraffin-embedded sections were obtained. The sections were then subjected to a DeadEnd™ Colorimetric TUNELSystem assay. The paraffin-embedded liver sections were re-treated to remove paraffin and fixed again in 4% paraformaldehyde. Following permeabilization, the sections were labeled with a terminal transferase reaction mix and bound to streptavidin horseradish peroxidase (HRP). The sections were then incubated with chromogen HRP and subsequently treated with diaminobenzidine and counterstained lightly with hematoxylin. Brown nuclei with nuclear condensation in the stained cells were considered to be TUNEL positive. Hepatocyte apoptosis in the liver sections was quantified by counting the number of TUNEL-positive cells in a random microscopic low-power field (magnification, ×400).

### Determination of lipid peroxidation and antioxidant enzyme activities

#### Malondialdehyde (MDA)

The lipid peroxide content in the liver was determined by quantifying the thiobarbituric acid reactive substances as previously described (21). Briefly, 0.5 ml supernatant was mixed with 1.5 ml thiobarbituric acid, 1.5 ml acetic acid (pH 3.5), 0.2 ml sodium dodecyl sulfate and 0.5 ml distilled water, and the samples and standards were heated to 100°C for 1 h. The absorbance was measured at 532 nm on a spectrophotometer. Commercially available MDA was used as a standard.

#### Superoxide dismutase (SOD)

SOD activity in the tissue homogenate was measured following the method previously described by Beauchamp and Friedrich (22), with slight modifications. The reaction mixture (0.8 ml), containing 100 μmol/l xanthine, 100 μmol/l EDTA, 25 μmol/l nitroblue tetrazolium (NBT) and 50 mmol/l Na_2_CO_3_ (pH 10.2), was added to 0.1 ml liver homogenate. After 10 min preincubation at room temperature, the reaction was initiated by the addition of 0.1 ml xanthine oxidase (0.05 U/ml), and the absorbance at 560 nm was recorded every 30 sec for 5 min. A standard curve for SOD activity was assayed spectrophotometrically as the inhibition of the photochemical reduction of NBT at 560 nm.

#### GSH-peroxidase (GSH-Px)

GSH-Px activity was determined as previously described, with slight modifications (23). The liver tissue was homogenized (1:10) in 75 mmol/l phosphate buffer (pH 7.0). The homogenate was then centrifuged at 20,000 × g for 25 min, and the supernatant was aspirated and assayed for total cytosolic GSH-Px activity. GSH-Px activity was assayed in a 3-ml cuvette containing 2.0 ml 75 mmol/l phosphate buffer (pH 7.0). The following solutions were then added: 50 μl glutathione (60 mmol/l), 100 μl glutathione reductase solution (30 U/ml), 50 μl NaN_3_ (0.12 mol/l), 100 μl Na_2_EDTA (15 mmol/l), 100 μl reduced nicotinamide adenine dinucleotide phosphate (NADPH; 3.0 mmol/l) and 100 μl cytosolic fraction. The reaction was initiated by the addition of 100 μl 7.5 mmol/l H_2_O_2_, and the conversion of NADPH to NADP was monitored by recording the change in the absorbance at 340 nm at 1-min intervals for 5 min. The GSH-Px activity was expressed as the quantity of reduced NADPH (in nanomoles) oxidized to NADP per minute per milligram of protein, with a molar extinction coefficient for NADPH at 340 nm of 6.22×10^6^.

#### Catalase

Catalase activity was determined as previously described (23). The liver tissue was homogenized (1:10) in 50 mmol/l potassium phosphate buffer (pH 7.4), and the homogenate was centrifuged at 40,000 × g for 30 min. Approximately 50 μl supernatant was added to a 3 ml-cuvette containing 2.95 ml hydrogen peroxide (19 mmol/l) in 50 mmol/l potassium phosphate buffer (pH 7.4). Changes in absorbance at 240 nm were recorded continuously for 5 min. Catalase activity was expressed as units per milligram of protein.

#### Measurement of total glutathione (GSSG+GSH) levels (reduced and oxidized)

The concentration of GSSG+GSH in the liver was measured using the glutathione reductase/5,5′-dithiobis-(2-nitrobenzoic acid) (DTNB) recycling assay (24). The rate of 5′-thio-2-nitrobenzoic acid formation was measured at an absorbance of 412 nm and was proportional to the sum of GSH+GSSG present. Liver tissue was homogenized in 5% sulfosalicylic acid, and the homogenate was centrifuged for 10 min at 10,000 × g. The supernatant was stored at 4°C until assayed. GSSG alone was measured by treating the sulfosalicylic acid supernatant with 2-vinylpyridine and triethanolamine. The solution was vigorously mixed, and the final pH of the solution was adjusted to between six and seven. After 60 min, the samples were assayed as described above in the DTNB-GSSG reductase recycling assay. The GSH values were calculated as the difference between the total (GSSG+GSH) and GSSG concentrations. The values are reported in GSH equivalents and expressed as micromoles per gram of tissue.

#### Hepatocyte preparation

Hepatocytes were isolated using a two-step collagenase perfusion method. Following mechanical disruption of the liver capsule, liver cells were collected in Williams’ Medium E and serially filtered (30-, 50- and 80-mesh) through an 85-ml Cellector (Bellco Biotechnology, Vineland, NJ, USA) tissue sieve. Between 10×10^6^ and 25×10^6^ cells were obtained from a single mouse liver.

#### Measurement of intracellular ROS

The fluorescent probe 2′,7′-dichloro-dihydro-fluorescein diacetate (DCFH-DA) is converted by intracellular esterases to DCFH, which is then oxidized by ROS to highly fluorescent DCF. The ROS Detection Reagent assay (Invitrogen Life Technologies, Carslbad, CA, USA) was performed in accordance with the manufacturer’s instructions. Hepatocytes were washed twice with cold phosphate-buffered saline (PBS) and incubated in Dulbecco’s Modified Eagle’s Medium containing 10 μM DCFH-DA (Invitrogen Life Technologies). Following centrifugation at 800 × g for 5 min and two washes with cold PBS, the fluorescence intensity of each group was determined using flow cytometric analysis (Becton-Dickinson, Franklin Lakes, NJ, USA) at excitation and emission (ex/em) wavelengths of 485/528 nm, respectively.

#### Assessment of the mitochondrial membrane potential (Δψ)

The mitochondrial membrane potential was assessed using the fluorescent dye JC-1 (Invitrogen Life Technologies), a lipophilic cationic dye that selectively enters mitochondria and reversibly changes color from green to red when the Δψ increases (25). Therefore, the ratio of the red to green fluorescent intensity of the cells reflects the Δψ. Suspended hepatocytes were incubated with JC-1 (200 μl) for 20 min at 37°C followed by two washes with PBS to remove the excess dye. Fluorescence was then measured using a flow cytometer (Becton-Dickinson) with ex/em wavelengths of 530/580 nm (red), and 485/530 nm (green).

#### Preparation of mitochondrial fraction

Mitochondria were isolated using conventional differential centrifugation from the liver of mice that had fasted overnight. The livers were homogenized in 250 mM sucrose, 1 mM ethylene glycol tetraacetic acid (EGTA) and 10 mM HEPES buffer (pH 7.2). The mitochondrial suspension was washed twice in the same medium containing 0.1 mM EGTA, and the final pellet was resuspended in 250 mM sucrose. The final protein concentration was 80–100 mg/ml, as measured by the Biuret method, using bovine serum albumin as the protein standard.

#### Mitochondrial swelling

The swelling experiments were performed as previously described by Beavis *et al* (26) using a standard medium containing 125 mM sucrose, 10 mM HEPES buffer (pH 7.2), 2.5 mM succinate and 4.0 mM rotenone at 25°C. The final volume used was 1.0 ml, and the protein concentration was ~0.5 mg/ml. Changes in absorbance at 520 nm were monitored in a thermostatically controlled Hitachi U 2000 spectrophotometer (Hitachi, Ltd., Tokyo, Japan).

#### Statistical analysis

Data analysis was performed using SPSS^®^ statistical software version 11.0 (SPSS Inc., Chicago, IL, USA). The data are expressed as the mean ± standard error of the mean from ≥12 independent experiments. Each treatment was performed in triplicate culture wells. The differences between the means of each group were tested using a one-way analysis of variance followed by the Student-Newman-Keuls test to compare between multiple groups. P<0.05 was considered to indicate a statistically significant difference.

## Results

### Taurine improves the liver-to-body ratio, as well as the ALT and AST levels, without affecting iron accumulation

Serum and hepatic iron levels were significantly increased in all iron-overloaded mice, regardless of taurine supplementation. To investigate whether liver injury and dysfunction were caused by iron overload, the liver-to-body weight ratio (%) and the levels of serum ALT and AST, which are important markers of dysfunction, were analyzed. Iron-overloaded mice showed a 1.9-fold increase in the liver-to-body weight ratio, and a 4.5- and 3.7-fold elevation in the serum ALT and AST levels, respectively. However, treatment with taurine was found to suppress these changes (Table I).

### Taurine prevents apoptosis in iron-overloaded mice

**Figure 1:**
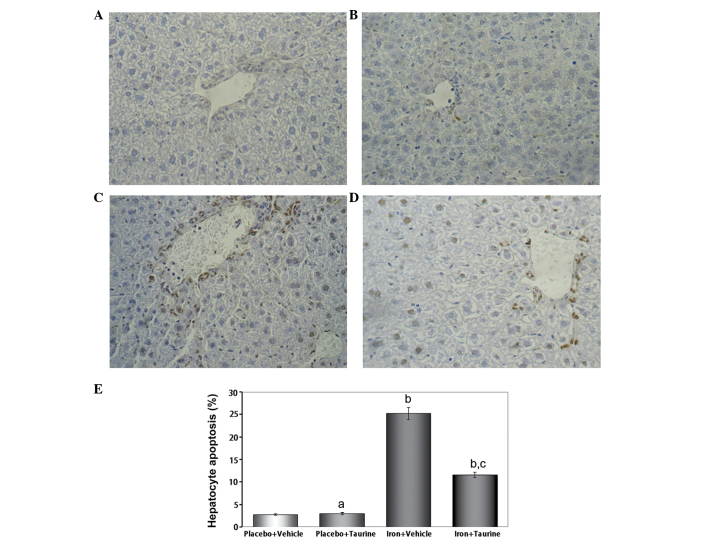
Effect of taurine on hepatocyte apoptosis in iron-overloaded mice. TUNEL-positive cells were apoptotic. (A–D) Liver sections from the different treatment groups: (A) placebo + vehicle, (B) placebo + taurine, (C) iron + vehicle and (D) iron + taurine (magnification, ×400). (E) Quantitative analysis of hepatocyte apoptosis expressed as the percentage of TUNEL-positive nuclei among the hepatocytes. Data are presented as the mean ± standard error of the mean (n=12). ^a^P>0.05 vs. the placebo + vehicle group; ^b^P<0.01 vs. the placebo + vehicle and placebo + taurine groups and ^c^P<0.01 vs. the iron + vehicle group. TUNEL, terminal deoxynucleotidyl transferase-mediated dUTP nick end labeling.

In mice that did not receive iron treatment, only a few TUNEL-positive hepatocytes were identified; however, numerous TUNEL-positive hepatocytes were observed in the iron-overloaded animals. Taurine supplementation significantly reduced the total number of TUNEL-positive hepatocytes to 11.6±0.62% of the total cell count (Fig. 1).

### Taurine ameliorates the decreased activities of antioxidant enzymes and increased lipid peroxidation induced by iron overload

**Figure 2:**
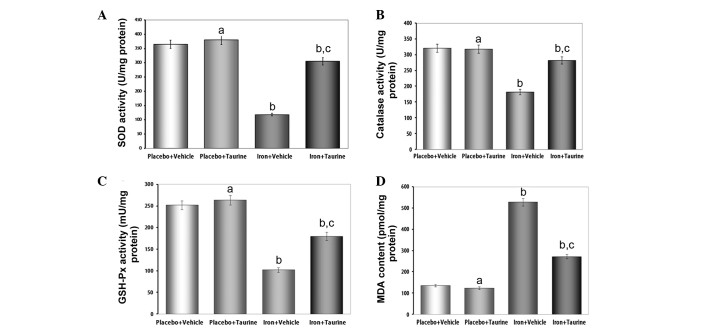
Effect of taurine on the activities of the antioxidant enzymes and lipid peroxidation in mice injected with iron over a 13-week period. (A–C) Activity of (A) SOD, (B) catalase, (C) GSH-Px and (D) MDA content. Data are presented as the mean ± standard error of the mean (n=12). ^a^P>0.05 vs. the placebo + vehicle group; ^b^P<0.01 vs. the placebo + vehicle and placebo + taurine groups and ^c^P<0.01 vs. the iron + vehicle group. SOD, superoxide dismutase; GSH-Px, glutathione-peroxidase; MDA, malondialdehyde.

SOD, catalase and GSH-Px are the important antioxidant enzymes present in the body that provide a defensive mechanism against free radical-mediated oxidative damage. Excess iron levels affect the activities of these enzymes as a result of the overproduction of ROS. The antioxidant activity of SOD is mediated by a dismutation reaction, in which SOD scavenges highly reactive superoxide radicals and converts them to oxygen molecules and less reactive H_2_O_2_ molecules (27). Catalase then further metabolizes the H_2_O_2_ into water and O_2_. The intracellular redox status is also maintained by the activity of GSH-Px in the presence of glutathione. GSH-Px aids in the decomposition of H_2_O_2_ and other organic hydroperoxides into non-toxic products (28). It has been observed in a number of previous studies that liver injury is associated with a reduction in the activities of these antioxidant enzymes (29,30). Therefore, in the present study, the effect of iron overload on the activities of different antioxidant enzymes was investigated. It was found that there was a significant decrease in the activity of the enzymes analyzed; however, treatment with taurine increased the activities of SOD, catalase and GSH-Px in the hepatic tissue of the mice (Fig. 2).

Lipid peroxidation is believed to be one of the most important parameters of oxidative stress and is measured by estimating the MDA concentration, a lipid peroxidation end product. In the present study, increased levels of MDA were observed in the iron-overloaded livers of experimental animals; however, taurine treatment was effective in preventing the extreme iron-induced alterations. These results suggest that the protective effects of taurine in the liver are correlated with the reduction in iron-induced oxidative stress.

### Taurine protects GSH levels in iron-overloaded mice

**Figure 3:**
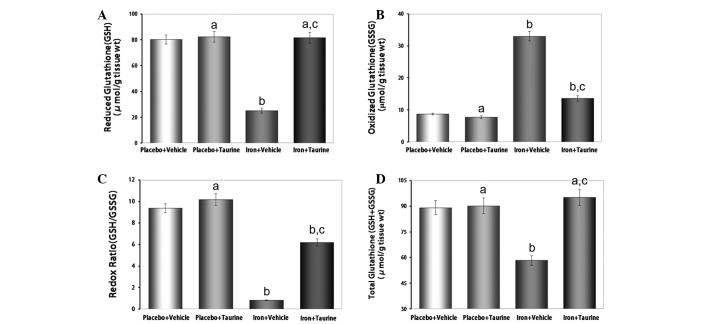
Effect of taurine on hepatic (A) GSH and (B) GSSG levels, (C) the redox ratio (GSH/GSSG) and (D) GSH+GSSG levels in mice injected with iron over a 13-week period. Data are presented as the mean ± standard error of the mean (n=12). ^a^P>0.05 vs. the placebo + vehicle group; ^b^P<0.01 vs. the placebo + vehicle and placebo + taurine groups and ^c^P<0.01 vs. the iron + vehicle group. GSH, glutathione; GSSG, glutathione disulfide.

Reduced glutathione, a ubiquitous tripeptide thiol, is an important intracellular metabolite that acts as an antioxidant and provides a secondary line of defense against intracellular free radicals and peroxides generated by oxidative stress (31). The reduced state of the cell is maintained by a high GSH/GSSG ratio. Owing to the potent antioxidant properties of taurine, it was hypothesized that taurine had the potential to preserve this high GSH/GSSG ratio in conditions of increased oxidative stress (32). It was observed that taurine supplementation in iron-overloaded mice almost entirely prevented the decrease in GSH (P<0.01) and increase in GSSG (P<0.01) levels, thereby partially protecting the redox ratio and normalizing the GSH+GSSG levels (Fig. 3).

### Taurine inhibits ROS generation in iron-overloaded mice

**Figure 4:**
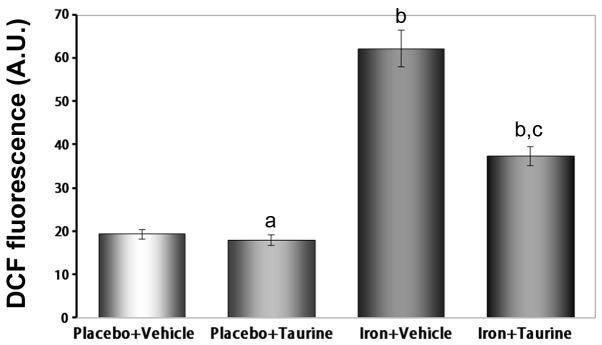
Effects of taurine on hepatic intercellular reactive oxygen species generation in mice injected with iron over a 13-week period. Data are presented as the mean ± standard error of the mean (n=12). ^a^P>0.05 vs. the placebo + vehicle group; ^b^P<0.01 vs. the placebo + vehicle and placebo + taurine groups and ^c^P<0.01 vs. the iron + vehicle group. DCF, dichloro-dihydro-fluorescein.

It is well established that iron induces toxicity in the liver, as iron is primarily stored in the liver and produces ROS (33). Therefore, the intracellular ROS levels were measured using a DCFH-DA assay. The ROS levels were observed to be significantly increased in the iron-treated animals compared with those in the untreated animals (P<0.01). This indicates that supplementation with taurine significantly prevents ROS formation induced by iron overload (Fig. 4).

### Taurine inhibits mitochondrial swelling in iron-overloaded mice

**Figure 5:**
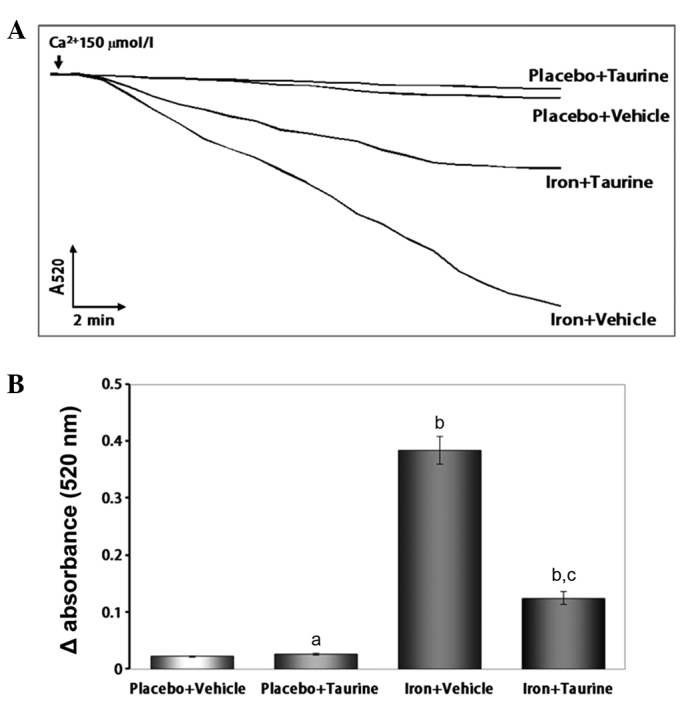
Effect of taurine on hepatocyte mitochondrial swelling in mice injected with iron over a 13-week period. Data are presented as the mean ± standard error of the mean (n=12). (A) Ca^2+^-induced mitochondrial swelling in iron-overloaded mice, with or without taurine supplementation. (B) Quantitative analysis of mitochondrial swelling. ^a^P>0.05 vs. the placebo + vehicle group; ^b^P<0.01 vs. the placebo + vehicle and placebo + taurine groups and ^c^P<0.01 vs. the iron + vehicle group.

Iron-induced damage to the inner mitochondrial membrane may be assessed using classic swelling techniques, which monitor the net influx of the osmotic support associated with a non-specific increase in membrane permeability. It was shown that iron induced mitochondrial swelling, indicated by the decrease in the absorbance of the mitochondrial suspension at 520 nm. However, taurine inhibited this swelling process (P<0.01) (Fig. 5).

### Taurine attenuates the loss of the Δψ in iron-overloaded mice

**Figure 6:**
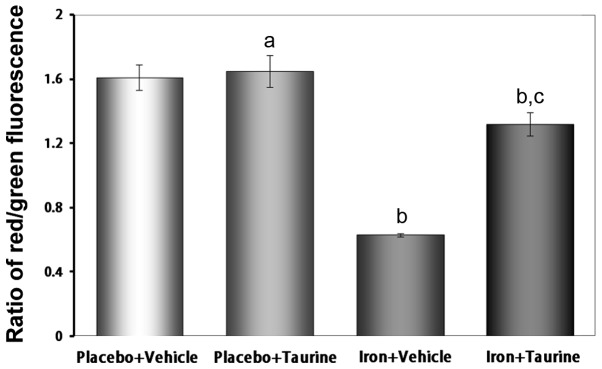
Effect of taurine on the mitochondrial membrane potential in mice injected with iron over a 13-week period. Data are presented as the mean ± standard error of the mean (n=12). ^a^P>0.05 vs. the placebo + vehicle group; ^b^P<0.01 vs. placebo + vehicle and placebo + taurine groups and ^c^P<0.01 vs. the iron + vehicle group.

The loss of the Δψ is an early indicator of apoptosis. The unique fluorescent cationic dye JC-1 was used to measure the Δψ in the mitochondria of hepatocytes. In healthy cells, where the Δψ is normal, JC-1 accumulates as aggregates in the mitochondrial matrix, where it emits red fluorescence. In apoptotic and dead cells, where the Δψ is lost, JC-1 exists in a monomeric form and emits green fluorescence. Therefore, the red to green fluorescence intensity ratio may be used to evaluate the Δψ. As shown in Fig. 6, the iron plus vehicle group exhibited a decrease in the ratio of red to green fluorescence intensity (P<0.01), indicating a loss of the Δψ. However, treatment with taurine significantly attenuated the loss of the Δψ (P<0.01).

## Discussion

Previous studies in humans and animals have shown that iron overload is a risk factor for liver damage (34). Although oxidative stress is considered to be a major cause of hepatotoxicity induced by iron overload, the underlying mechanism remains unknown. In the present study, mice that were injected with iron exhibited marked symptoms of iron toxicity, including an elevated liver-to-body weight ratio, increased levels of AST and ALT in the plasma and serum and intracellular iron deposition in the liver, concurrent with increased apoptosis.

Free radical production and oxidative stress play key roles in the initiation and progression of iron overload hepatotoxicity (35). In the livers of iron-overloaded mice, marked increases in ROS and MDA (an end product of lipid peroxidation) levels were observed, as well as a depletion of GSH and GSH+GSSG levels. These findings were indicative of increased oxidative damage. Free radical-induced lipid peroxidation leads to cellular dysfunction. Clinically, the AST and ALT levels in the plasma represent biomarkers for liver function (36). In the present study, the serum AST and ALT levels in the mice were significantly elevated following iron overload, suggesting an iron overload-related injury to the liver.

Taurine is a conditionally essential amino acid that contains a sulfonic acid group and has several physiological roles (37,38). Increasing evidence has shown that taurine exerts strong protective effects due to its antioxidant characteristics (17,39,40). In the present study taurine levels in the liver were increased to investigate whether this prevented iron overload-induced hepatic damage, despite the fact that taurine has no effect on iron deposition. This rationale was based on the finding that there is an increased demand for taurine in instances of oxidative stress. The results showed that taurine supplementation was associated with the protection of liver function in iron-overloaded mice. The protective effects of taurine occurred concurrently with a reduction in apoptosis, suggesting that taurine was able to decrease the toxic effects of iron. The protective effects of taurine have been previously associated with its antioxidant properties. Furthermore, taurine has been shown to reduce iron-mediated lipid peroxidation and increase the activities of antioxidant enzymes (41).

Consistent with the antioxidant properties of taurine observed in the present study, a marked reduction in MDA formation was observed in the iron-overloaded mice. The potent antioxidant properties of taurine are additionally associated with increased antioxidant enzyme activity. SOD, catalase and GSH-Px are the key cellular antioxidant enzymes that defend against oxidative stress. Evidence shows that the activities of these antioxidant enzymes are decreased when cells and tissues are subjected to oxidative stress (42,43). The antioxidant effects attributed to taurine may be associated with its sulfur moiety, and the modulation of GSH and GSH levels by taurine is critical in the cellular defense against oxidative stress (44,45). During oxidative stress, increased cellular demand leads to the depletion of GSH and the accumulation of GSSG. As a result, a shift in the redox state occurs and the cell function becomes impaired. In the present study, taurine supplementation completely prevented the iron-induced depletion in the GSH levels while protecting the redox ratio. These findings elucidate a possible mechanism underlying the actions of taurine and may explain its pleiotropic and beneficial effects following an increase in oxidative stress (42,43).

Hepatocyte damage mediated by oxidative stress is associated with the disruption of the Δψ (46). Mitochondria are important organelles that have a vital role in apoptosis (47). The mitochondrial permeability transition pore (mPTP) is a multiprotein complex that can form large, nonselective pores in the inner mitochondrial membrane (48). ROS are one of the most important factors stimulating the opening of the mPTPs. If the mPTPs are constantly open, mitochondrial swelling can occur, resulting in the rupture of the outer mitochondrial membrane and culminating in apoptosis. The present study demonstrated that overproduction of ROS in iron-overloaded mice increased the mitochondrial membrane permeability, induced the opening of the mPTPs, and resulted in mitochondrial swelling and depolarization (49). Therefore, the collapse in Δψ may be a consequence of the oxidative stress in hepatocytes. Taurine treatment led to a reduction in the liver ROS levels due to its strong ROS scavenging and antioxidant properties, as previously mentioned. Reduced ROS levels prevented the mPTPs from opening and the subsequent mitochondrial swelling, which blocked Δψ depolarization.

In conclusion, the murine model of an iron-overloaded liver used in the present study demonstrated that taurine supplementation has beneficial effects on liver function and apoptosis, with marked reductions in oxidative stress induced by iron overload. Due to the benefits provided and the absence of toxicity with taurine supplementation, increased dietary intake of taurine represents an important nutritional modification that could be a useful therapeutic alternative to reduce the hepatic toxicity induced by iron overload in such diseases as hemosiderosis.

## Figures and Tables

**Table I:** Effect of taurine on the serum and hepatic iron concentration, liver-to-body weight ratio, serum levels of ALT and AST and hepatic taurine levels in iron-injected mice.

Parameter	Placebo + vehicle	Placebo + taurine	Iron + vehicle	Iron + taurine
Serum iron concentration (μmol/l)	34.64±1.32	32.80±1.41^a^	420.36±15.42^b^	401.2±13.82^b^,^c^
Hepatic iron concentration (mg/g dry weight)	0.068±0.003	0.062±0.003^a^	1.042±0.026^b^	1.021±0.028^b^,^c^
Liver-to-body ratio (mg/g)	48.2±1.9	46.8±2.1^a^	92.4±4.1^b^	61.5±2.5^b^,^d^
ALT (U/l)	50.81±1.52	48.83±1.65^a^	228.31±9.42^b^	125.06±5.33^b^,^d^
AST (U/l)	106.20±3.58	113.42±3.91^a^	395.13±14.22^b^	216.42±8.23^b^,^d^
Taurine level (μmol/g)	30.03±1.51	49.16±2.63^e^	28.52±1.62^a^	47.38±2.85^d^,^f^

Data are expressed as the mean ± the standard error of the mean (n=12).

a P>0.05 vs. the placebo + vehicle group;

b P<0.01 vs. the placebo + vehicle and placebo + taurine groups;

c P>0.05 vs. the iron + vehicle group;

d P<0.01 vs. the iron + vehicle group;

e P<0.01 vs. the placebo + vehicle group and

f P>0.05 vs. the placebo + taurine group.

ALT, alanine transaminase; AST, aspartate transaminase.
